# Osr1 regulates hepatic inflammation and cell survival in the progression of non-alcoholic fatty liver disease

**DOI:** 10.1038/s41374-020-00493-2

**Published:** 2020-10-01

**Authors:** Yi Zhou, Zhimin Liu, Ernest C. Lynch, Leya He, Henghui Cheng, Lin Liu, Zhen Li, Jiangyuan Li, Lauren Lawless, Ke K. Zhang, Linglin Xie

**Affiliations:** 1 Department of Nutrition, Texas A&M University, College Station, TX 77843; 2 Tongji Hospital, Huazhong University of Science and Technology, Wuhan, Hubei 430030, China; 3 Department of Colorectal Surgery, The Sixth Affiliated Hospital of Sun Yat-sen University (Gastrointestinal and Anal Hospital of Sun Yat-sen Unversity), Guangzhou 510655, China; 4 Department of Statistics, Texas A&M University, College Station, TX 77843; 5 Center for Epigenetics & Disease Prevention, Institute of Biosciences & Technology, College of Medicine, Texas A&M University, Houston, TX

## Abstract

Odd-skipped related 1 (Osr1) is a novel tumor suppressor gene in several cancer cell lines. Non-alcoholic steatohepatitis (NASH) is considered as a high-risk factor for hepatocellular carcinoma (HCC). This study is aimed to investigate the novel role of Osr1 in promoting the progression of hepatic steatosis to NASH. Following 12 weeks of diethylnitrosamine (DEN) and high-fat diet (HFD), wildtype (*WT*) and Osr1 heterozygous (*Osr1*^*+/−*^) male mice were examined for liver injuries. *Osr1*^*+/−*^ mice displayed worsen liver injury with higher serum alanine aminotransferase levels than the *WT* mice. The *Osr1*^*+/−*^ mice also revealed early signs of collagen deposition with increased hepatic *Tgfb* and *Fn1* expression. There was overactivation of both JNK and NF-κB signaling in the *Osr1*^*+/−*^ liver, along with accumulation of F4/80+ cells and enhanced hepatic expression of *Il-1b* and *Il-6*. Moreover, the *Osr1*^*+/−*^ liver displayed hyperphosphorylation of AKT/mTOR signaling, associated with overexpression of *Bcl-2*. In addition, *Osr1*^*+/−*^ and *WT* mice displayed differences in the DNA methylome of the liver cells. Specifically, Osr1-responsible CpG islands of *Ccl3* and *Pcgf2,* genes for inflammation and macrophage infiltration, were further identified. Taken together, Osr1 plays an important role in regulating cell inflammation and survival through multiple signaling pathways and DNA methylation modification for NAFLD progression.

## Introduction

Over the past few decades, the prevalence of non-alcoholic fatty liver disease (NAFLD) has rapidly increased and affects 70–80% of the obese population. Non-alcoholic steatohepatitis (NASH) is a more severe type of NAFLD, characterized by hepatic steatosis and inflammation, with or without fibrosis (1–3). Patients with NASH have an increased risk of developing hepatic fibrosis, which can progress to hepatocellular carcinoma (HCC) and cirrhosis (1–3). Currently, NASH has become the leading cause of chronic liver disease in developed countries. In addition, NAFLD is also a risk factor for cardiovascular disease, independent of other metabolic factors (1–3). As a consequence, it is classified as a medical condition with high therapeutic needs.

The pathogenesis of NASH is considered to be multifactorial with several pathways involved, and can be described by a “two-hit” hypothesis. The “first hit” is classified by enhanced lipogenesis and lipolysis due to insulin resistance, which can then progress to lipotoxicity accompanied by oxidative stress, lipid peroxidation, mitochondrial dysfunction, and increased hepatic inflammation, comprising the “second hit” (4–6). Several signaling pathways have been found to contribute to the onset and progression of NASH. The insulin, MAPK, and JNK signaling pathways contribute to hepatic fat accumulation and cell injury in the pathogenesis of NAFLD in the rat (7–10). The AMPK and mTOR signaling pathways are known to participate in altering metabolic homeostasis (11, 12). In addition, the Toll-like receptor, JNK, and Nuclear factor-κB (NF-κB) signaling pathways promote the production of pro-inflammatory cytokines and chemokines, driving the innate immune system to mediate steatosis, inflammation, and fibrosis (13–15).

The *Osr1* gene encodes a putative transcription factor containing four C2H2-type zinc finger motifs (16). It was first reported to regulate the proliferation of cardiac precursors during embryonic heart development, and to mediate apoptosis during urogenital development (17–21). An Osr1 deletion results in embryonic lethality before E14.5 (17, 19, 21). Epigenetic regulation was found to control the expression of Osr1. Hypermethylated CpG islands associated with Osr1 transcription have been reported in lung and gastric carcinoma, and renal tumor cells (20, 22, 23). Osr1 has recently been reported as a novel tumor suppressor gene (22, 24–28), as well as a potential prognostic biomarker in gastric cancer (24). However, the role of Osr1 in the progression of NAFLD towards HCC development has yet to be determined.

In this study, a high-fat (HF) diet plus diethylnitrosamine (DEN) induced NASH model on Osr1 heterozygous male mice was adapted. To understand the role of Osr1 in NAFLD/NASH progression, hepatic fat accumulation and liver injury, along with the associated molecular differences of the Osr1 heterozygous mice versus the wildtype were evaluated.

## Materials and Methods

### Chemicals, Antibodies, and Reagents

Diethylnitrosamine (DEN) was purchased from Sigma Chemical Co. (St. Louis, MO, USA). Antibodies against IRS1, phospho-IRS-1(Ser1101), NF-κB, phospho-NF-κB, phospho-AKT(Ser473), phospho–AKT (Thr308), JNK, phospho-SAPK/JNK (Thr183/Tyr185), phospho-p38MARK (Thr180/Tyr182), phospho-p44/42 MARK (Erk1/2) (Thr202/Tyr204), mTOR, phospho-mTOR, and GAPDH were purchased from Cell Signaling Technology (USA). The bicinchoninic acid (BCA) protein assay kit and the phosphatase inhibitor tablets were purchased from Thermo Fisher Scientific (USA).

### Animals

Male *Osr1*^*+/−*^ and WT mice (C57BL/6 background), purchased from Jackson Laboratory, were treated with either chow diet (CD) or DEN and HFD from weaning to 12 weeks of age. Only male mice were included in the study due to their increased sensitivity to HFD treatment (29–31). The mice were then humanely euthanized, and blood and liver samples were collected for experiments. All mouse experiments were completed according to a protocol reviewed and approved by the Institutional Animal Care and Use Committee of Texas A&M University, in compliance with the USA Public Health Service Policy on Humane Care and Use of Laboratory Animals.

### Diet and treatments

Diets were purchased from Research Diets, LLC (New Brunswick, NJ, USA). The HFD (Cat. no. D12492) had an energy density of 5.157 kcal/g (60% fat, 20% carbohydrate, and 20% protein). The fat source is composed of 92% lard and 8% soybean oil. The concentrations of vitamins, minerals, and proteins were modified to ensure that these nutrients in the HF diet were equivalent to those in the chow diet on a per kilocalorie basis. Mice were injected intraperitoneally (i.p.) with 20–25 μg/g of DEN once at 21 days of age.

### Intraperitoneal injected glucose tolerance test (IPGTT)

At the end of week 12, offspring mice from each experimental group were fasted overnight for 12h and then subjected to an IPGTT early the next morning. Glucose tolerance tests were conducted with a dose of 2.0 g/kg body weight. The tail vein blood glucose levels were measured with an automated glucometer (Bayer, Elkhart, IN) at baseline and 15, 30, 60, and 120 minutes after the injection.

### Analysis of hepatic triglyceride (TG), serum alanine aminotransferase (ALT) activity and insulin concentration

Serums were collected and stored at −80°C until measured. Serum ALT activity was measured using an ALT Activity Assay kit (MAK052, Sigma-Aldrich, USA) according to the manufacturer’s instructions. The plasma concentrations of insulin were determined using an insulin enzyme-linked immunosorbent assay (ELISA) commercial kit (RayBiotech, USA) according to the manufacturer’s instructions. The hepatic TG content was measured using the Triglyceride Assay Kit (Abcam, Cambridge, UK) according to the manufacturer’s instructions.

### Histopathology evaluation

Mouse livers were collected, fixed in 10% buffered formalin for 48h, and then were subjected to histopathological processing, followed by paraffin embedding. Sections were taken at 5 microns thick and at least five serial sections per sample were collected for histological analysis. Sections were subjected to hematoxylin-eosin staining for granuloma counting and steatosis evaluation, and Masson trichrome staining (Newcomer supply 9179A, USA) for collagen fiber observation.

For Immunofluorescent (IF) staining and Immunohistochemistry (IHC) staining, the slides of liver sections with 5 microns in thickness were deparaffinized with xylene before staining. First antibody Osr1, purchased from Santa Cruz (sc-376529, California) were used at concentration of 1:50, and F4/80 from ThermoFisher Scientific were used at 1:200. Secondary antibodies of IF staining used were: F(ab’)2-Goat anti-Mouse IgG-Alexa 594 (Catlog#A11020, 1:800 dilution) and Goat anti-Rabbit IgG-Alexa 488 (Catlog#A31628, 1:800 dilution) from Thermo Fisher Scientific. Chemical reagent used were DAPI (Catlog#F6057) from Sigma-Aldrich (Oakville, ON, Canada). Primary antibody incubation took place in a humidified box overnight at 4℃. Secondary antibody incubation took place at room temperature for 30 minutes in a dark box. Incubation was stopped by washing the slides with PBS. After staining by DAPI, IF slides were observed by fluorescence microscope under corresponding wave length. For IHC staining, slides were treated followed the protocol of VECTASTAIN ABC-HRP Kit for IHC (Fisher NC9213702). Slides were reacted with ImmPACT DAB Peroxidase substrate (Fisher NC9567138) at the final step and scanned by ScanScope FL from Leica.

### RNA extraction and RT-PCR

Total RNA was extracted using TRIzol reagent (Thermo Fisher Scientific, USA), and the concentration was determined in triplicate using a NanoDrop ND-1000 spectrophotometer (Thermo Scientific). Total mRNA (1ug) was amplified and reverse transcribed using ReadyScript®cDNA Synthesis Mix (Sigma-Aldrich, USA), and qPCR was performed using CFX384™ Real-Time System (BIO-RAD, USA) with the CFX Manager 3.1software. Primers used for RT-PCR analysis are shown in Supplementary Table 1.

### Reduced-Representation Bisulfite Sequencing (RRBS)

RRBS was performed on the liver samples of three *WT* and *Osr1*^*+/−*^ littermate mice exposed to HFD and DEN for 12 weeks, according to the manual’s instruction of Ovation RRBS Methyl-Seq System 1–16 (NuGEN Technologies, Inc., San Carlos, CA). Briefly, liver genomic DNA was extracted using Quick-DNA Universal Kit (ZYMO Research, USA). Genomic DNA was digested with MspI for 1 hour, and ligated to methylated adapters with ligation adaptor mixes and ligation enzyme mix. The reaction was then subjected to final repair using the final repair enzyme mix at 60°C for 10 minutes, followed by 10 minutes at 70°C. Bisulfite conversion was performed using QIAGEN EpiTect Fast DNA Bisulfite Kit (QIAGEN, USA). RRBS libraries were amplified by PCR with the amplification enzyme mix and subjected for sequencing on an Illumina HiSeq 4000 at Texas A&M AgriLife Genomics and Bioinformatics Core (College Station, TX).

### Bioinformatics analysis of RRBS

Bisulfite sequencing reads from RRBS were aligned and CpG methylation was analyzed using Bismark (v0.16.3) (32). Differential methylation regions (DMR) were analyzed using an in-house program that was developed based on machine learning algorithm, mean shift, and regularized t-test. The mean shift algorithm was used to locate the regions with densely methylated CpG sites. This customized mean shift algorithm was constructed on kernel density estimation (KDE). A Gassuain kernel with 50 bp bandwidth was used to quantify the weight of nearby points. The program then iteratively shifts to the next CpG site for identifying the nearest peak on the KDE curve. Differential methylation was assessed by a regularized t-test method as defined below.

$$t=\frac{m_{t}-m_{c}}{s_{e}}$$

where m_t_ and m_c_ are the means for the treatment and control groups, respectively, and s_e_ is the estimated standard error which is calculated as follows:

$$s_{e}=s_{p}\sqrt{\frac{1}{n_{c}}+\frac{1}{n_{t}}}$$

$$s_{p}=\sqrt{\frac{\left(n_{c}-1\right)s_{c}^{2}+\left(n_{t}-1\right)s_{t}^{2}}{n_{c}+n_{t}-2}}$$

where s_c_^2^ and s_t_^2^ are the estimated standard deviations for the control and treatment groups, respectively.

Differentially methylated regions (DMRs) located within 5 kb upstream to 1 kb downstream of transcription start sites were considered for further analysis for their associations with gene expression.

### Statistical analysis

Differences between the WT and *Osr1*^*+/−*^ groups were analyzed by one-way ANOVA. For the longitudinal data, such as body weight and food consumption, a linear mixed model was used for the analysis of repeated measures with each individual mouse as a random effect. All analyses were carried out by using SAS JMP software (SAS Institute Inc., Cary, NC, USA) and R statistical programming language.

## Results

### Osr1 is expressed in hepatocytes and macrophages in liver.

**Figure 1.**
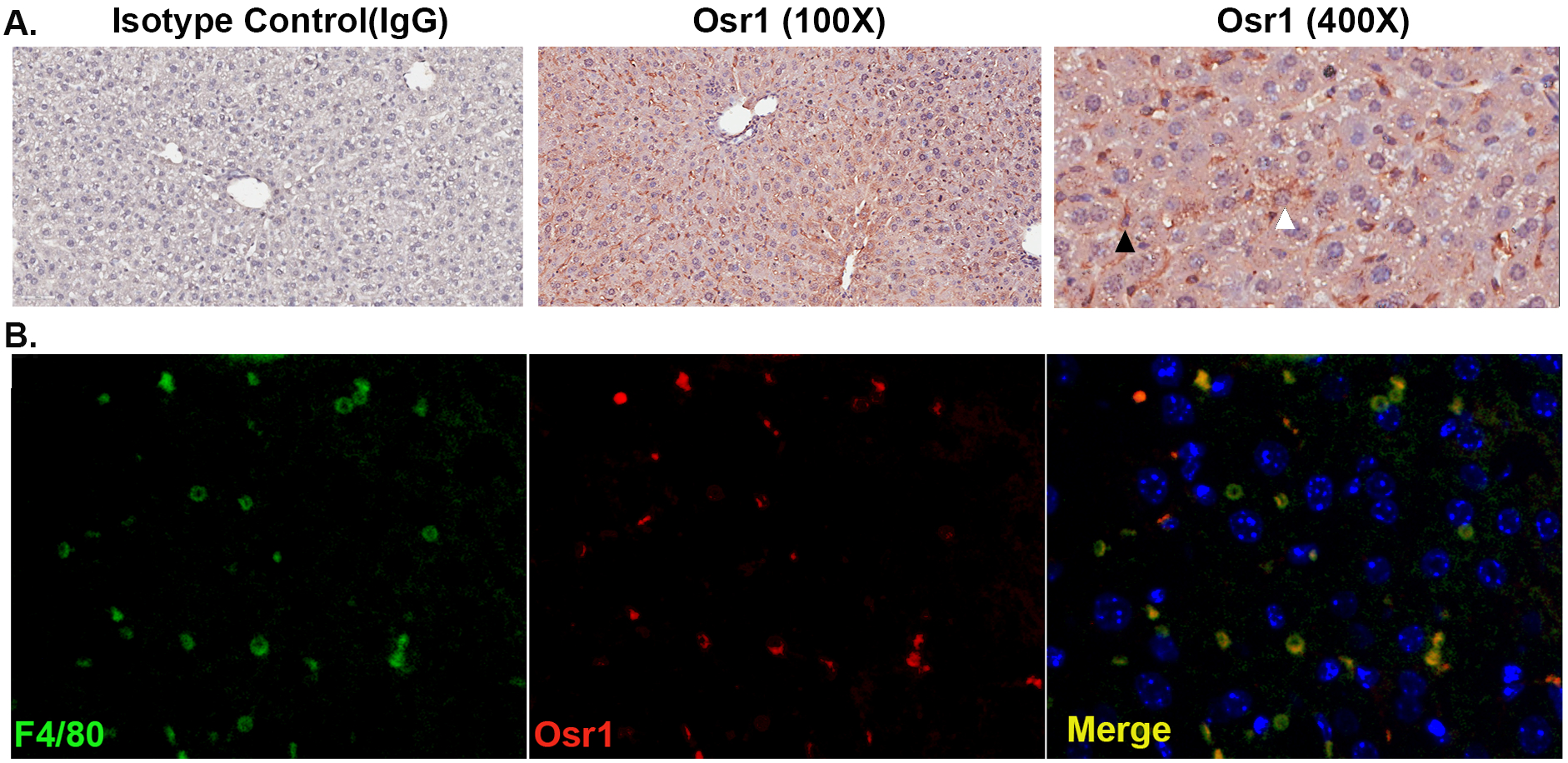
Osr1 is expressed in hepatocytes and macrophages in the liver. (A) IHC staining of Osr1 on WT liver tissue. White arrow head indicates weak Osr1 expression in the hepatocytes. Black arrow head indicates strong Osr1 expression in liver macrophages. (B) Co-IF staining of Osr1, F4/80 and DAPI on WT liver tissue.

Osr1 is expressed in the developing heart and the intermediate mesoderm during mouse embryonic development. Its mRNA expression is lower, but detectable in the liver (24). Osr1 expression in the liver of *WT* mice was observed by IHC staining. Osr1 staining was prominently detected in two types of cells. The cytosol of hepatocytes displayed weak, but positive staining for Osr1 in a scattered format (Figure 1A, white arrowhead), while spindle shaped, non-hepatocytes, suspected to be macrophages, displayed a strong expression of Osr1 (Figure 1A, black arrowhead).Thus, co-IF staining was performed using anti-F4/80 and anti-Osr1. The colocalization of F4/80 and Osr1 indicated that Osr1 is highly expressed in liver macrophages (Figure 1B).

### Hepatic expression of Osr1 in the *Osr1*^*+/−*^ mouse is about half that in the *WT* mouse.

**Figure 2.**
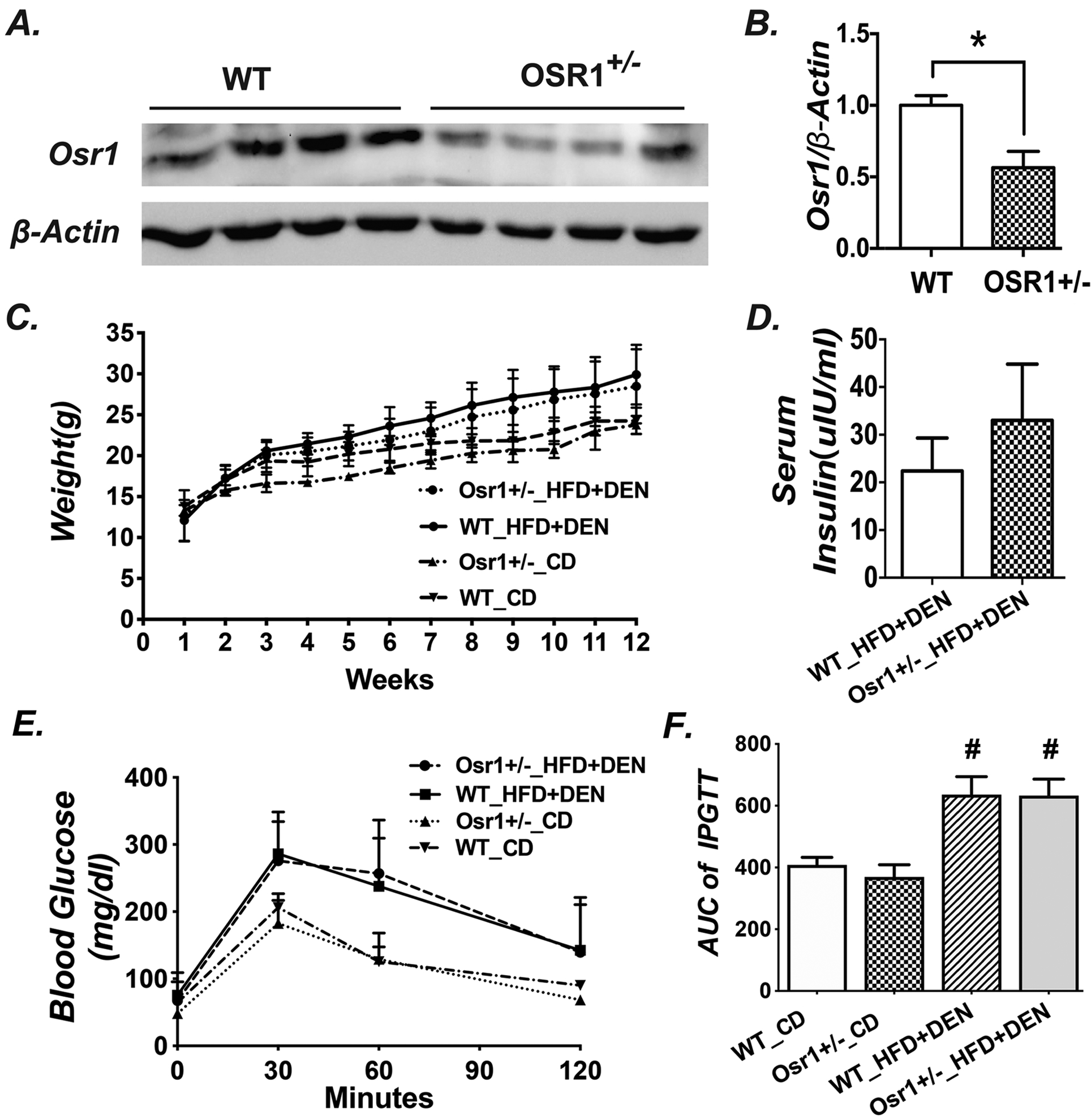
The body weight and glucose tolerance were not different between *Osr1*^*+/−*^ and *WT* mice during the experimental period. (A) Western blot results of Osr1 in the livers of *Osr1*^*+/−*^ and *WT* mice. (B) Quantification of Osr1 expression in the liver measured by Western Blots. (C) Body weight of *Osr1*^*+/−*^ and *WT* mice during the 12-week treatment period. (D) Serum insulin level of *Osr1*^*+/−*^ and *WT* mice exposed to HFD and DEN treatment for 12-weeks. (E) IPGTT results tested at 12 weeks of age, upon HFD and DEN treatment. (F) Area under the curve of IPGTT Data is presented as Mean ± SE, n=8. * *P*<0.05, ** *P*<0.01,*** *P*<0.001 vs. WT group.

Western blots were performed on the liver samples of the *Osr1*^*+/−*^ and *WT* male mice (Figs. 2A and B). Western blot confirmed Osr1 expression in the liver of the *WT* mice. As expected, the Osr1 expression level in *Osr1*^*+/−*^ mice was only half of that in the WT liver.

### Osr1 downregulation neither affected body weight gain, nor glucose tolerance

The average body weight was not different between the *WT* and the *Osr1*^*+/−*^ groups when offered the chow diet or the HFD and DEN treatment. However, both the *WT* and *Osr1*^*+/−*^groups continuously gained weight faster on the HFD and DEN treatment than the groups on CD (Fig. 2C). After a 12-week treatment on HFD and DEN, the serum insulin levels of the *Osr1*^*+/−*^ group showed an increasing trend versus the *WT* groups, although the difference is not statistically significant (Fig.2D). The IPGTT was also performed at the end of week 12. Consistent with the insulin levels, the fasting glucose levels of the *Osr1*^*+/−*^ group was similar to the *WT* mice no matter which diet was provided (Fig.2E). Both the *Osr1*^*+/−*^ and *WT* groups responded alike to the glucose challenge (Figs.2E and F).

### Upon HFD and DEN treatment, the *Osr1*^*+/−*^ mice had more severe liver injury compared to the *WT* mice.

**Figure 3.**
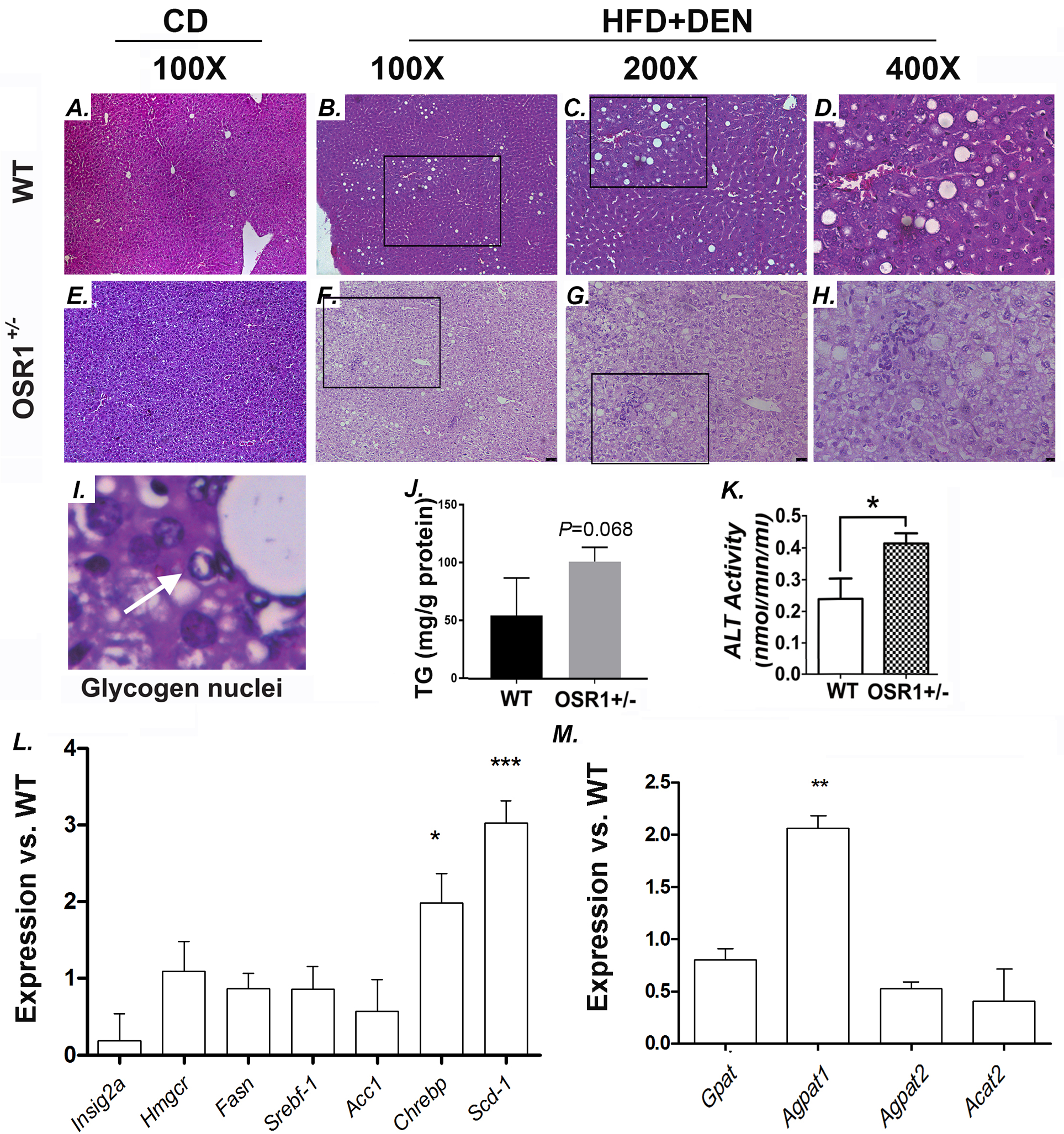
*Osr1*^*+/−*^ mice displayed worsen liver injury than WT upon HFD and DEN treatment. (A-H) Histology of the liver (HE staining). (I) Visible glycogen nuclei were only observed in the *Osr1*^*+/−*^ liver upon HFD and DEN treatment. (J)Hepatic TG content of *Osr1*^*+/−*^ and WT livers upon HFD and DEN treatment. (K) Serum ALT level of *Osr1*^*+/−*^ and WT livers upon HFD and DEN treatment was measured as described in Methods. (L and M) Expression of key genes involved in lipid metabolism was measured by RT-PCR in *Osr1*^*+/−*^ and WT livers upon HFD and DEN treatment. Data is presented as Mean ± SE, n=4. * *P*<0.05, ** *P*<0.01,*** *P*<0.001 vs. WT group.

On the CD, both the *Osr1*^*+/−*^ and *WT* mice displayed normal liver histology at 12 weeks (Fig. 3A and E). However, lipid drop accumulation in the livers of both the *Osr1*^*+/−*^ and *WT* mice was observed following the HFD and DEN treatment (Fig.3). The *Osr1*^*+/−*^ mice displayed more severe liver injury, likely a borderline NASH, evidenced by obvious hepatic steatosis, a moderate number of foci, hepatocellular ballooning, and the presence of visible glycogenated nuclei (Fig.3I). Interestingly, these histopathological changes were not observed in the *WT* liver (Figs.3A–D). Hepatic triglyceride (TG) content of *Osr1*^*+/−*^ mice was higher than the WT mice with marginal significance (P=0.068) (Fig. 3J). Serum ALT levels were measured and found to be significantly higher in the *Osr1*^*+/−*^ mice (Fig.3K). In addition, important genes involved in lipogenesis, such as *Chrebp* and *Scd-1* (Fig. 3L), and genes involved in phospholipid synthesis, such as *Agpat1,* were expressed more prominently in the *Osr1*^*+/−*^ mice (Fig. 3M). These results suggest that *Osr1*^*+/−*^ mice experienced more severe liver damage when compared to the *WT* mice upon HFD and DEN.

Although obvious NASH was not observed after the 12-week treatment of HFD and DEN, a NASH CRN Scoring System was used to evaluate the NAFLD progression in the *Osr1*^*+/−*^ and *WT* mice (Table 1) (33). From 9 *Osr1*^*+/−*^ mice 5 (56%) scored higher than “2” and 2 (22%) scored higher than “4”, while only 1 out of 8 *WT* mice scored higher than “2” or “4” (12.5%). The *Osr1*^*+/−*^ mice scored marginally higher than the WT group (2.89 vs. 1.13, P=0.0767). When one *WT* mouse scored “5”, considered an outlier, was removed from the group, the *WT* group was given an average score of 0.57, which was significantly lower than the 2.89 score of the *Osr1*^*+/−*^ group (P=0.0080). To be noted, the lobular inflammation score and the ballooning score of the *Osr1*^*+/−*^ group were both significantly higher than those of the *WT* group.

### Upon HFD and DEN treatment, the *Osr1*^*+/−*^ mice displayed early signs of fibrogenesis.

**Figure 4.**
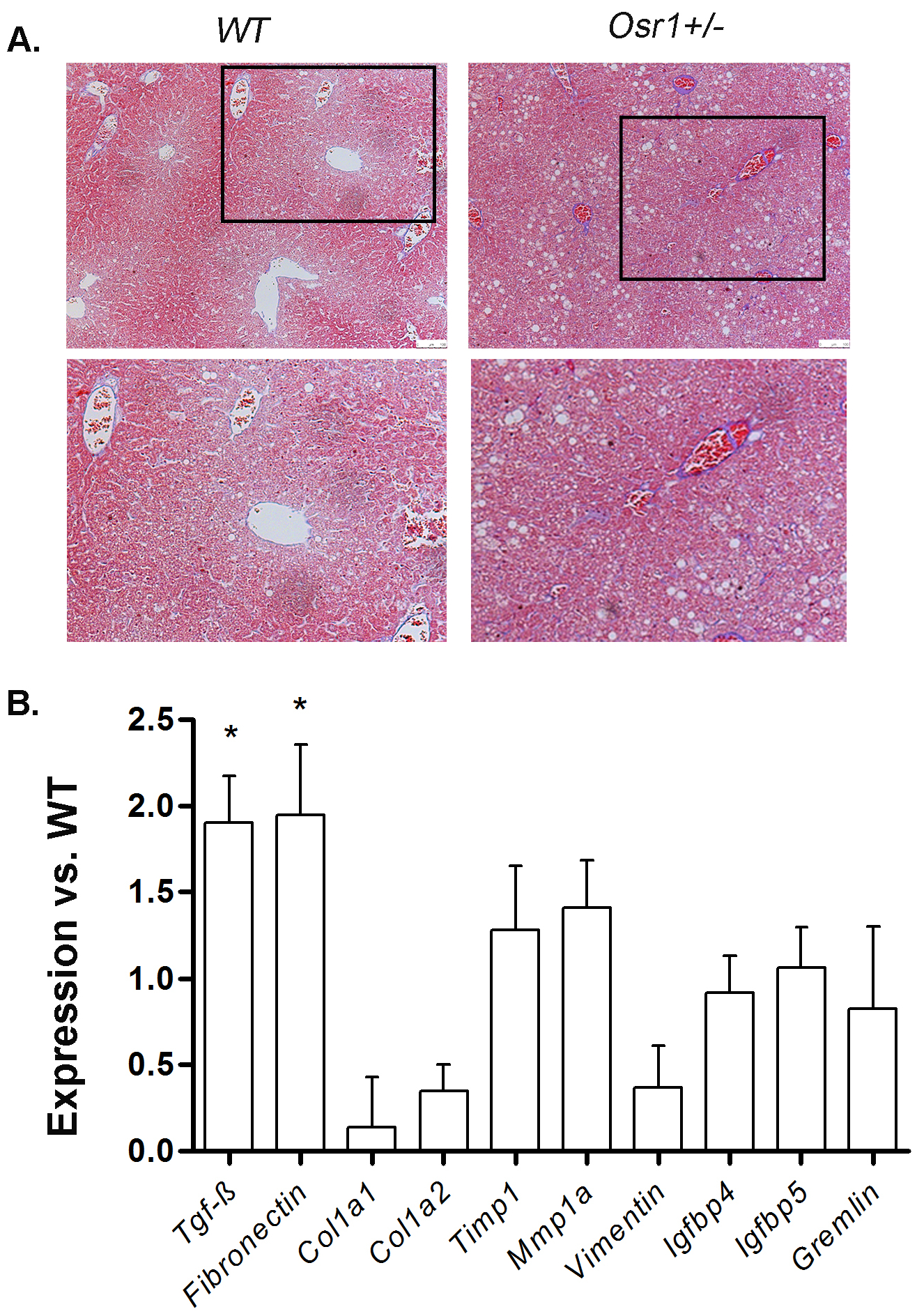
The *Osr1*^*+/−*^ liver displayed more collagen deposition than WT liver upon HFD and DEN treatment. (A) Masson-Trichrome staining of the *Osr1*^*+/−*^ and WT livers upon HFD and DEN treatment. (B) Expression of key genes involved in fibrogenesis was measured by RT-PCR in *Osr1*^*+/−*^ and WT livers upon HFD and DEN treatment. Data is presented as Mean ± SE, n=4. * *P*<0.05 vs. WT group.

Trichrome staining did not display any sign of fibrosis in the WT mice, while the *Osr1*^*+/−*^ mice showed deposition of collagen fibers along perisinusoids or between the central vein and portal tracts, indicating the initiation of fibrosis (Fig. 4A). The *Osr1*^*+/−*^ mice also displayed significantly elevated expression of *Tgfβ* and *Fn1* (Fig. 4B). These results suggest that reduced Osr1 expression, along with a high fat diet and DEN treatment, promotes liver injury and fibrosis onset.

### Upon HFD and DEN treatment, the *Osr1*^*+/−*^ mice showed increased hepatic inflammation.

Although the *Osr1*^*+/−*^ mice did not respond abnormally to the glucose challenge, the relationship between the severely injured liver and desensitized insulin signaling remains in question. Insulin signaling was detected by measuring the expression levels of Irs-1 and p-Irs1in the liver (Fig. 1S). However, the expression of Isr1 nor p-Isr1 at the Ser^301^, Ser^612^, and Ser^636/639^ residues were affected in *Osr1*^*+/−*^ mice in comparison to the *WT*.

The association of Osr1 levels and AMPK activity in the liver was then examined. Similar to the insulin signaling results, (Fig.2S) no expression changes of AMPK-α, -β, and Acc, nor phosphorylation alterations of these subunits were observed in the *Osr1*^*+/−*^ mice.

**Figure 5.**
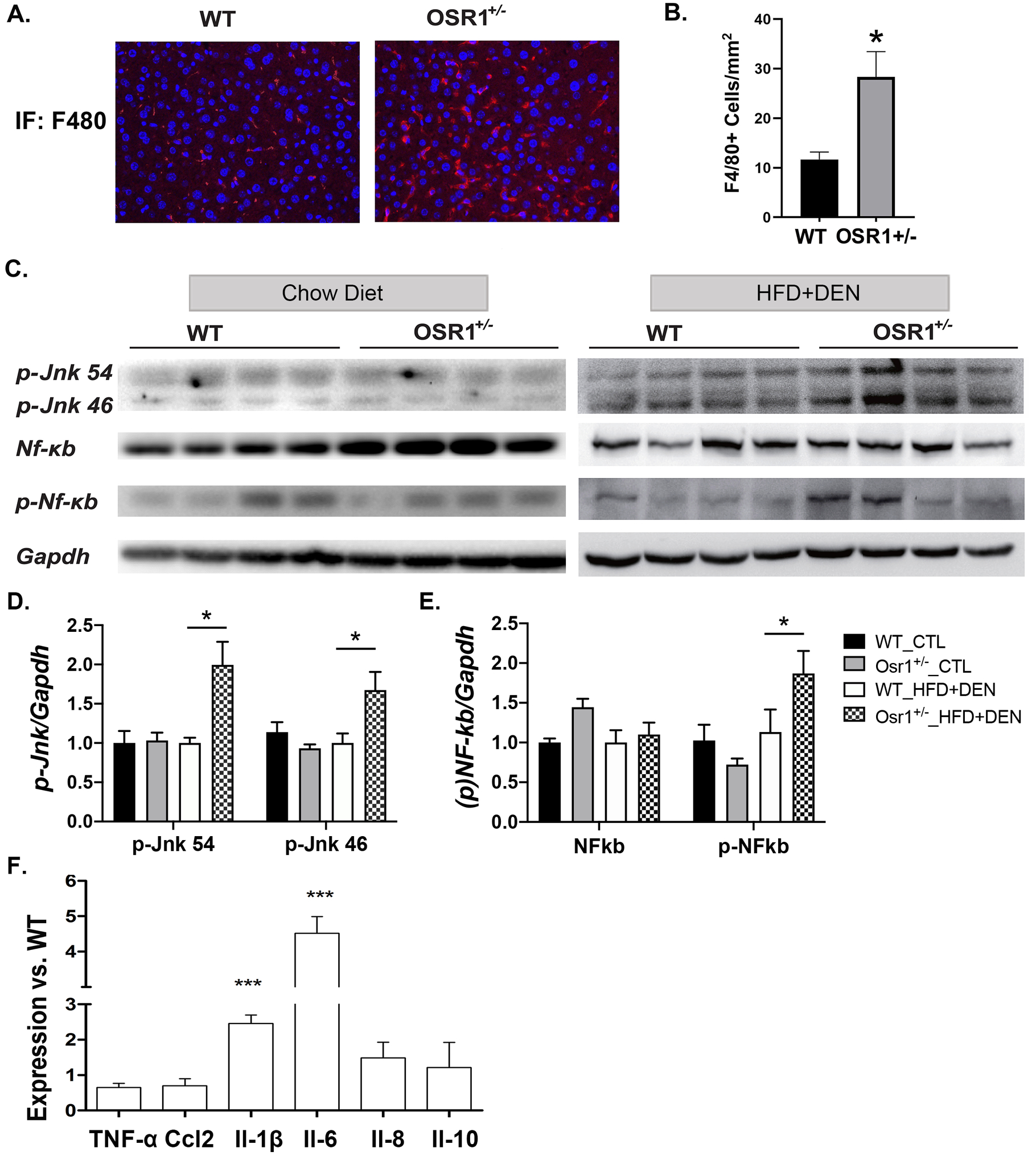
The *Osr1*^*+/−*^ liver displayed more inflammation than the WT liver upon HFD and DEN treatment. (A) IF staining of F4/80 on liver tissues showed increased macrophage accumulation in the liver of *Osr1*^*+/−*^ mice. (B) There were more F4/80+ cells accumulation in the *Osr1*^*+/−*^ liver than the WT liver upon HFD and DEN treatment. (C-E)Western blot of p-JNK (46/54) and p-NF-κB showed higher expression in *Osr1*^*+/−*^ mice exposed to HFD and DEN. (F) Expression of key genes involved in inflammation was measured by RT-PCR in *Osr1*^*+/−*^ and WT livers upon HFD and DEN treatment. Data is presented as Mean ± SE, n=4. * *P*<0.05, ** *P*<0.01,*** *P*<0.001 vs. WT group.

According to the “two hit” hypothesis, hepatic inflammation triggers the “second hit” for NAFLD progression (34). Compared to the *WT* mice under HFD and DEN treatment, the *Osr1*^*+/−*^ mice displayed increased hepatic macrophage infiltration, which could be observed by F4/80 staining (Fig. 5A) and evaluated by counting the number of F4/80 positive cells (Fig. 5B). The relationship between Osr1 and the regulation of inflammatory signaling pathways, such as NF-κB and JNK activity, was then measured in the liver. (Fig.5C). Osr1 deficiency did not change the hepatic expression of p-JNK54/46, NF-κB and p-NF-κB under chow diet (Figs. 5C–E). However, the *Osr1*^*+/−*^ mice significantly enhanced hepatic phosphorylation levels of both p54 JNK and p46 JNK when compared to the *WT* controls exposed to HFD and DEN (Figs.5C and D). The *Osr1*^*+/−*^ mice also exhibited a higher level of phosphorylated NF-κB (Figs.5C–E). Consistently, the hepatic expression of *Il-1β* and *Il-6* was significantly higher in the *Osr1*^*+/−*^ mice exposed to HFD and DEN (Figs.5F). There was no observable difference between the expression of *Tnf-α, Ccl-2, Il-8* and *Il-10* in the *Osr1*^*+/−*^ and the *WT* mice.

### *Osr1*^*+/−*^ mice displayed enhanced hepatic Akt/m-TOR activity and altered expression of apoptotic genes.

**Figure 6.**
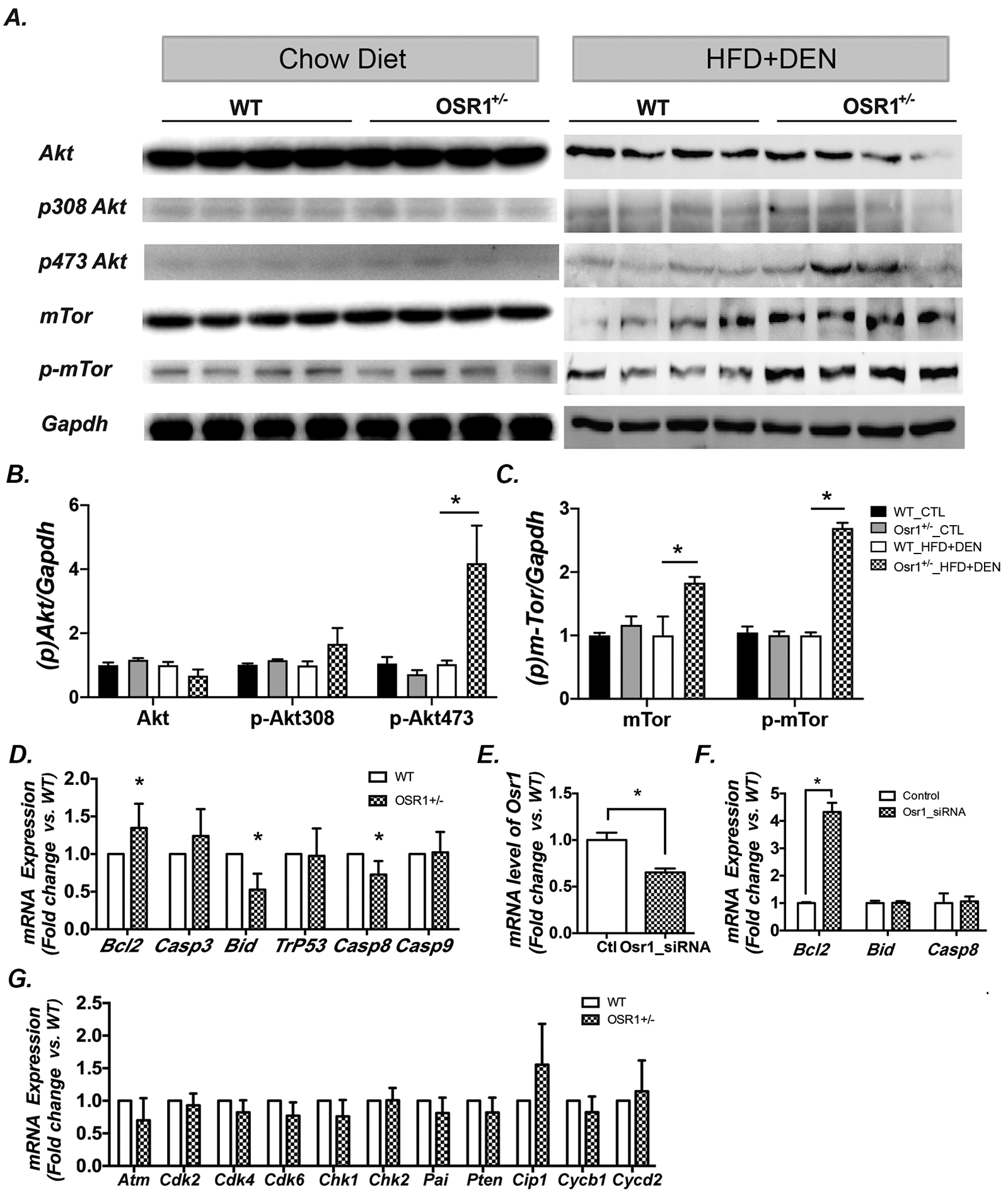
*Osr1*^*+/−*^ mice displayed enhanced hepatic Akt/m-TOR activity and altered expression of apoptotic genes. (A-C)Western blot of liver tissues showed a higher expression of p-Akt at Ser473 in *Osr1*^*+/−*^ mice, but not at Ser308, without effecting basal levels of Akt (6A & B). Higher protein expression of mTOR and p-mTOR in *Osr1*^*+/−*^ mice exposed to HFD and DEN were detected by Western blot (6A, C, & D). (D)Expression of key genes involved in apoptosis was measured by RT-PCR in *Osr1*^*+/−*^ and WT livers upon HFD and DEN treatment. Expression of the anti-apoptotic gene, *Bcl-2*, was up-regulated in *Osr1*^*+/−*^ mice, and pro-apoptotic genes, *Bid* and *Casp8*, were down-regulated. (E-F) Knockdown of Osr1 using Osr1-siRNA on HEK293T cells caused an up-regulation of *Bcl-2*, but not *Bid* or *Casp8*. (G) Expression of key genes involved in cell proliferation was measured by RT-PCR in *Osr1*^*+/−*^ and WT liver upon HFD and DEN treatment. None of the cell proliferation related genes were found to have expression alterations between *Osr1*^*+/−*^ and WT mice. Data of figures is presented as Mean ± SE, n=4. * *P*<0.05 vs. WT group

Osr1 has been previously reported as a tumor repressor gene in gastric cancer (24). Thus, the current study, adapting a HFD and DEN treatment, was designed to induce abnormal cell proliferation and apoptosis. Although this study failed to induce hepatocellular carcinoma due to short-term treatment, the correlation of Osr1 expression levels with cell proliferation and survival, in concurrence with the HFD and DEN treatment, was still worth investigating. Activation of the Akt/mTOR signaling pathway, which is known to regulate cell death, (35–37) was then detected (Fig.6). Osr1 deficiency did not change the hepatic expression of Akt, p-Akt, mTOR and p-mTOR under chow diet (Figs. 6A–C). However, exposed to HFD and DEN, the *Osr1*^*+/−*^ mice displayed hyperphosphorylation of Akt at Ser^473^, but not at Ser^308^, without basal Akt protein level changes (Figs.6A and B). The *Osr1*^*+/−*^ mice also displayed increased expression of both mTOR and phosphorylated mTOR, suggesting overactivation of the Akt/mTOR signaling pathway (Figs.6A and C).

Next, the mRNA levels of the key apoptotic genes, including *Bcl2, Casp3, Bid, Trp53, Casp8* and *Casp9*, were measured by real-time PCR. The anti-apoptotic gene, *Bcl2,* was markedly increased in the *Osr1*^*+/−*^ mice, while the pro-apoptotic genes, *Bid* and *Casp8*, were downregulated (Fig. 6D). The expression of Osr1 was then knocked down in HEK293 cells by RNAi (Fig. 6E). With the lower Osr1 expression, there was significantly elevated mRNA levels of *Bcl-2,* while the expressions of *Bid* and *Casp8* remained unchanged. These results suggest that *Osr1* heterozygous mice display an enhanced ability of cell survival (Fig. 6F).

The effect of the diminished expression of Osr1 on cell proliferation was then addressed. The mRNA levels of genes involved in cell proliferation, such as *Atm, Cdk2, Cdk4, Cdk6, Chk1, Chk2, Pai, Pten, Cip1, Cycb1,* and *Cycd2*, were measured. The expression of these cell proliferation genes in the livers of the *Osr1*^*+/−*^ and *WT* mice showed no significant changes (Fig. 6G).

### *Osr1*^*+/−*^ mice displayed differences in the DNA methylome of their hepatocytes

**Figure 7.**
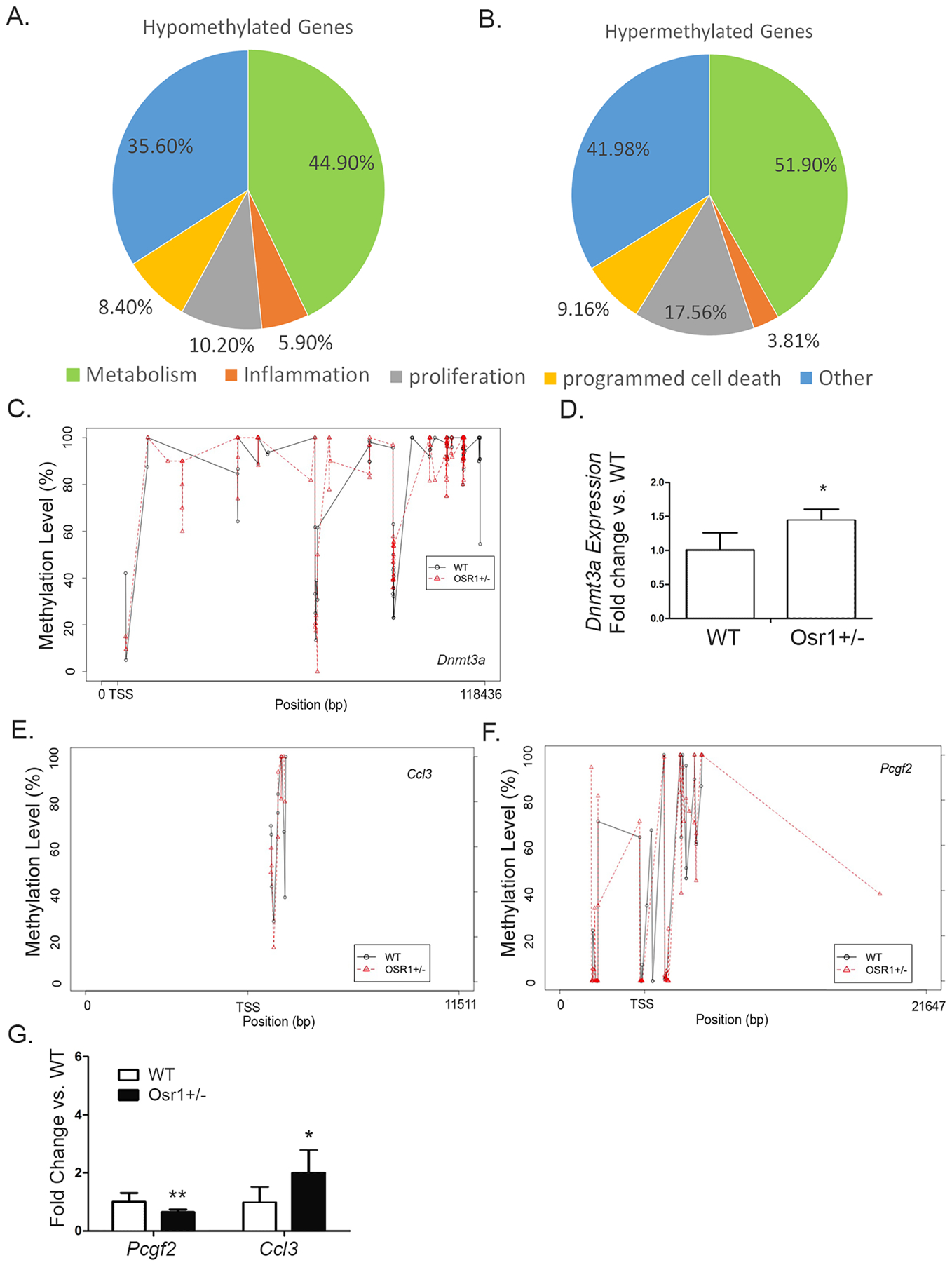
Upon HFD and DEN, *Osr1*^*+/−*^ mice displayed differences in the DNA methylome of their hepatocytes. (A and B) Liver methylomes were generated at single-base resolution by RRBS in both WT and *Osr1*^*+/−*^ mice. The RRBS data identified 233 genes with 144 and 109 hyper- or hypomethylated genomic sites (CpG island) in the *Osr1*^*+/−*^ and WT livers, respectively. These genes were categorized by the biological processes of metabolism, inflammation, proliferation, programmed cell death, and other by Gene Ontology (GO) analysis. (C) With RRBS analysis, several CpG sites within 5kb upstream of TSS and 1kb downstream of TES of *Dnmt3a* were found (6C). (D) There was a marginally significant increase of *Dnmt3a* expression in the *Osr1*^*+/−*^ liver compared to the WT measured by RT-PCR (E and F) Several methylation sites of *Ccl3* and *Pcgf2* were identified by RRBS. (G)Expression of *Ccl3* and *Pcgf2* was detected by RT-PCR. Data of figures is presented as Mean ± SE, n=4. * *P*<0.1, ** *P*<0.05 vs. Wildtype group

Because expression of Osr1 is controlled by epigenetic regulation and hypermethylation of CpG islands (22), the role of Osr1 as a regulator of DNA methylation was explored. To address this question, both *WT* and *Osr1*^*+/−*^ liver methylomes were generated at single-base resolution by RRBS. As expected, most of the hypomethylated CpGs resided in the CpG islands proximal to the transcription start sites (TSSs). The methylation sites located between 5kbp upstream of the TSS and 5kbp downstream of the transcription end sites (TESs) were subjected to bioinformatic analysis. The RRBS data identified 233 genes with 144 and 109 hyper- or hypomethylated genomic sites (CpG islands) in the *Osr1*^*+/−*^ and *WT* livers, respectively. These genes were categorized according to their biological role of either metabolism, inflammation, proliferation, programmed cell death, or other by Gene Ontology (GO) analysis. The majority of the genes, 51.9% hypermethylated and 44.9% hypomethylated, were related to metabolism (Figs. 7A and B). Approximately 5.9%, 10.2%, and 8.4% hypomethylated genes were characterized as inflammatory, proliferative, and apoptotic genes, respectively (Fig. 7A). Roughly 3.81%, 17.56% and 9.16% hypermethylated genes were classified as inflammatory, proliferative, and apoptotic, respectively (Fig. 7B).

*Dnmt3a* encodes for DNA (cytosine-5)-methyltransferase 3A, which catalyzes the transfer of methyl groups to CpG sites in DNA. With RRBS analysis, several CpG sites within the 5kb upstream of TSS and 5kb downstream of TES were found (Figure 7C). Among these sites, a hypomethylated CpG site (P=0.0165) located 488bp downstream of TSS was identified. The methylation level at the site was 29.52% lower in the *Osr1*^*+/−*^ liver (Table 2). There was a marginally significant increase of *Dnmt3a* expression in the *Osr1*^*+/−*^ liver compared to the WT (Figure 7D, P<0.1).

### DNA methylation on *Ccl3* and *Pcgf2* was associated with *Osr1* levels

Among these genes, expression changes of *Ccl3* and *Pcgf2* between the *WT* and *Osr1*^*+/−*^ livers were found to be associated with the methylation changes within their transcription regulatory regions. Several methylation sites of both genes were identified by RRBS (Fig. 7E and F). One genomic locus of *Ccl3*, located 740bp downstream of the TSS, displayed a 24.55% decrease in the methylation level (Fig. 7E and Table 2). The expression of *Ccl3* exhibited a 1-fold increase in the *Osr1*^*+/−*^ group when compared to the *WT* (Fig. 7G, P<0.1 with marginal significance). The methylation level of *Pcgf2* at 1843bp downstream of the TSS was 23.53% higher in the *Osr1*^*+/−*^ group (Fig. 7F and Table 2), which is consistent to its decreased expression in the *Osr1*^*+/−*^ liver (Fig. 7G, P<0.05).

## Discussion

In the past three years, a new role of Osr1 as a novel tumor suppressor has been reported in various types of cancer, including lung, gastric and renal carcinoma (22, 24–28). It has also been found to be a potential prognostic biomarker in gastric cancer (24). However, its role in HCC onset and progression remains unknown. NASH is considered a high-risk factor for HCC. DEN is an acute hepatotoxin and carcinogen studied in mouse models. A single application of DEN at 10–90 mg/kg body weight is sufficient to induce hepatic tumors in rodents (38, 39). In the current study, a single dose of DEN at 25μg/g body was given to the C57BL/J6 mice at weaning. This protocol successfully induced premalignant lesions after 24 weeks and HCC-like tumors after 42 weeks on C57BL/J6 mice (39), demonstrating the efficiency for DEN to cause liver injury. In addition to the DEN treatment, a 60% HFD was provided to the mice for 12 weeks to promote NASH development. In general, at least 12-week of HFD treatment is required to induce severe steatohepatitis on C57BL/J6 mice (40). In the current study, obvious pathological changes of NASH were not present, although the *Osr1*^*+/−*^ mice displayed borderline NASH diagnostic potential (score =2.89). This was evidenced by hepatic steatosis, with inflammation and ballooned hepatocytes (33). In this study, the *Osr1*^*+/−*^ mice displayed more advanced fatty liver disease, featured with more severe steatosis and hepatocellular ballooning, as well as increased macrophage infiltration. In addition, increased *Tgfb* and *Fn1* expression and deposition of collagen fibers between the central vein and portal tracts in the *Osr1*^*+/−*^ mice suggested the active progression towards NASH. This study provided novel evidence on the important role of Osr1 on NAFLD progression.

Development of HCC is known to undergo sequential histopathological changes, denoted as the hepatic inflammation-fibrosis-cancer (IFC) axis (41). Numerous studies have identified NF-κB as a potential master orchestrator of the IFC axis through the regulation of immune, fibrogenic, and oncogenic mediators. Powerful activation of NF-κB signaling induces the secretion of proinflammatory cytokines, including TNF-α and IL-6, which then promotes fibrosis in the hepatic immune system (41, 42). Strong activation of JNK has also been observed in the liver of mice on a high-fat diet (HFD), and JNK knockout mice were found to be protected from HFD-induced hepatocyte injury and steatosis (43, 44). Similar to NF-κB signaling, the activation of the JNK pathway contributes to the production of inflammatory cytokines, such as TNF-α, IL-1, and IL-6, which further induces hepatocellular injury, inflammation, and fibrosis (45, 46). Osr1 deficiency does not affect the NF-κB and JNK signaling under physiological conditions. However, macrophage infiltration, associated with the activation of NF-κB and JNK signaling, along with an overexpression of *Tnfα* and *Il-1β* were found in the *Osr1*^*+/−*^ mice exposed to HFD and DEN. These mice also displayed early signs of collagen deposition, suggesting an active progression of hepatic inflammation to fibrosis. These results suggest that Osr1 participates in the regulation of hepatic inflammation via NF-κB and JNK signaling. Interestingly, a recent study on tongue squamous cell carcinoma reported that Osr1 inhibited tumor cell migration and invasion by obstructing the NF-κB pathway (25, 26). To be noted, strong Osr1 expression was observed in the macrophages of the liver, highly suggesting an important role of Osr1 in the regulation of hepatic inflammation. Future studies using a transgenic mouse model specifically deleting Osr1 in the macrophages will disclose this potential role of Osr1 in NASH progression.

The PI3K/AKT/mTOR pathway is a major intracellular signaling pathway that regulates multiple cellular functions, including cell proliferation and apoptosis. Activation of this pivotal signaling pathway, resulting in the inhibition of apoptosis, is found in 30–50% of HCC cases (47). Although the treatment of our study did not induce HCC due to a short experimental period, it is known to be an effective method to cause HCC and over activate oncogenic signaling pathways (38). A universal precancerous feature is enhanced cell survival and proliferation. The transactivation of anti-apoptotic genes, such as *Bcl2*, provides an explanation for the inhibition of programmed cell death by NF-kB signaling (48). An intricate crosstalk between the NF-κB and JNK signaling pathways influences the cell’s decision between life and death (48, 49). In this study, the hyperactivation of the PI3K/AKT/mTOR, NF-κB, and JNK signaling pathways in the *Osr1*^*+/−*^ mice indicated an increase in cell survival upon DEN and HFD treatment. These findings support the evidence that decreased *Osr1* expression results in elevated expression of anti-apoptotic genes, such as *Bcl2* and *Bid*. Being protected from programmed cell death, the injured cells may undergo self-fixation and eventually become malignant (50). Future studies will adapt the same model with a longer experimental period to disclose the importance of Osr1 for cell survival though the regulation of multiple signaling pathways, including the PI3K/AKT/mTOR, NF-κB, and JNK, during HCC development.

Expression of *Osr1* is controlled by epigenetic regulation. Methylation of CpG islands in Osr1 coding regions has been documented in multiple cancer cell lines (22, 51). As a tumor suppressor gene, its role in epigenetic regulation may be elucidated. In this study, a marginally increased expression of *Dnmt3a* was reported, and a total of 253 CpG methylation sites were identified by RRBS analysis in the *Osr1*^*+/−*^ liver. These CpG sites are located within the regulatory region of 233 different genes that play important roles in regulating cell metabolism, inflammation, proliferation, and apoptosis. In addition, two important genes, *Ccl3* and *Pcgf2,* with their CpG methylation sites potentially under the regulation of Osr1, were identified. CCL3, also known as macrophage inflammatory protein-1α, is responsible for liver immune cell infiltration and therefore mediates experimental liver fibrosis (52). The increased expression and hypomethylation of *Ccl3* is consistent with the increased macrophage infiltration and inflammation in the *Osr1*^*+/−*^ liver. The *Pcgf2* gene negatively regulates the expression of different cytokines, chemokines, and chemokine receptors, and plays a role in decreasing immune cell migration (53). In this study, expression of *Pcgf2* was downregulated in the *Osr1*^*+/−*^ liver, and the hypermethylated CpG site was identified. These results suggest that Osr1 influences DNA methylation, and might act as a key regulator of the mechanism driving cancer development.

In summary, Osr1 plays an important role in regulating cell survival, cell inflammation, and macrophage migration in liver. Accordingly, this study has identified Osr1 as a novel repressor gene in the progression of NAFLD/NASH.

## Supplementary Material

1

## Figures and Tables

**Table 1.** NAS Score for Osr1 and WT group on HFD and DEN.

Sample ID	Group	Steatosis	Lobular Inflammation	Ballooning	Total Score	Score>2	Score>4
7639	Osr1	2	2	2	6	+	+
7641	Osr1	1	1	0	2	−	−
7644	Osr1	1	2	2	5	+	+
7645	Osr1	1	1	2	4	+	−
7558	Osr1	0	0	0	0	−	−
7559	Osr1	1	1	1	3	+	−
7562	Osr1	1	1	2	4	+	−
7564	Osr1	1	0	0	1	−	−
7566	Osr1	1	0	0	1	−	−
7640	WT	1	2	2	5	+	+
7642	WT	1	0	1	2	−	−
7648	WT	1	0	0	0	−	−
7651	WT	1	0	0	1	−	−
7557	WT	0	0	0	0	−	−
7560	WT	1	0	0	1	−	−
7561	WT	0	0	0	0	−	−
7565	WT	0	0	0	0	−	−
P value*		0.2419	0.0972	0.1623	0.0767	0.1784	0.5997
P value*,^#^		0.2001	0.0092	0.0381	0.0080	0.0174	0.1824

Note:

* For steatosis, inflammation, ballooning and total score, P value was calculated by student T-test. For NAS incidence (Score>2 or Score >4), P value was calculated by Chi-square test. P<0.05 is considered statistically significant.

# P value was calculated by removing sample #7640, which was considered an outlier.

**Table 2:** CpG methylation sites of *Dnmt3a, Ccl3* and *Pcgf2* identified by RRBS.

Gene	Chromosome	Site	P-value	Meth.Diff	Dist. to TSS
*Dnmt3a*	chr12	3891313	0.0165	29.52%	431
*Ccl3*	chr11	83648639	0.0318	24.55%	740
*Pcgf2*	chr11	97697542	0.0119	23.53%	1843
