# Supplementary material for: Osr1 regulates hepatic inflammation and cell survival in the progression of non-alcoholic fatty liver disease
Source: Lab Invest. Author manuscript; Available in PMC 2021 Apr 1. (PMC7987871; doi:10.1038/s41374-020-00493-2)
Supplement: 1 [file NIHMS1628942-supplement-1.pdf]

Supplementary information

Table 1S Primer sets used for RT-PCR

	Forward(5'-3')	*Reverse(5'-3')*
Osr1	*CCATCCCTGCAGCTTACCAA*	*GCATGAAGAGCGCTGAAACC*
Osr1_human	*CGTTCTCAGCTGCTCCTGGTT*	*GTTTTGCTGCCCATTTCGGTA*
Chrebp	*CCAGCAGGGAAGGGGATTTC*	*AGAGCTGTTCGCACCATCG*
Scd-1	*AACACCCCGATAGCAATATCCA*	*AGAGAACTGGAGACGGGAGT*
Agpat1	*CTGGTTCGTTCGGTCCTTCA*	*CCATGAGACCCATCATGTCCA*
Tgf-β	*TCGCTTTGTACAACAGCACC*	*ACTGCTTCCCGAATGTCTGA*
Fn1	*TGCTCGTGAATCCTCTTGAACA*	*AACCTGTTTCCAATGGGCCT*
Il-β	*AAATACCTGTGGCCTTGGGC*	*CTTGGGATCCACACTCTCCAG*
Il-6	*CCAGAGATACAAAGAAATGATGG*	*ACTCCAGAAGACCAGAGGAAA*
Tnf-α	*AGCCGATGGGTTGTACCT-TG*	*ATAGCAAATCGGCTGACG-GT*
Ccl-2	*AGCACCAGCCAACTCTCACT*	*CGTTAACTGCATCTGGCTGA*
Il-8	*ATGCCTCTCCATTTCCTGCT*	*CATGGGGAAAGAGGCTCTGA*
Il-10	*TGAATTCCCTGGGTGAGAAG*	*TCACTCTTCACCTGCTCCACT*
Bcl-2	*GTCGCTACCGTCGTGACTTC*	*CAGACATGCACCTACCCAGC*
Casp3	*TGGTGATGAAGGGGTCATTTATG*	*TTCGGCTTTCCAGTCAGACTC*
Bid	*GCCGAGCACATCACAGACC*	*TGGCAATGTTGTGGATGATTTCT*
Trp53	*CTCTCCCCCGCAAAAGAAAAA*	*CGGAACATCTCGAAGCGTTTA*
Casp8	*TGCTTGGACTACATCCCACAC*	*TGCAGTCTAGGAAGTTGACCA*
Casp9	*TCCTGGTACATCGAGACCTTG*	*AAGTCCCTTTCGCAGAAACAG*
Bcl-2_human	*CTGGTGGGAGCTTGCATCAC*	*ACAGCCTGCAGCTTTGTTTC*
Bid_human	*ACTGGTGTTTGGCTTCCTCC*	*ATTCTTCCCAAGCGGGAGTG*
Casp8_human	*CAGCAAAGGGGAGGAGTT*	*CTTCAAAGGTCGTGGTCAAA*
Atm	*GATCTGCTCATTTGCTGCCG*	*GTGTGGTGGCTGATACATTTGAT*

Cdk2	*CCTGCTTATCAATGCAGAGGG*	*TGCGGGTCACCATTTCAGC*
Cdk4	*ATGGCTGCCACTCGATATGAA*	*TCCTCCATTAGGAACTCTCACAC*
Cdk6	*GGCGTACCCACAGAAACCATA*	*AGGTAAGGGCCATCTGAAAACT*
Chk1	*GTTAAGCCACGAGAATGTAGTGA*	*GATACTGGATATGGCCTTCCCT*
Chk2	*TGACAGTGCTTCCTGTTCACA*	*GAGCTGGACGAACCCTGATA*
Pai	*TTCAGCCCTTGCTTGCCTC*	*ACACTTTTACTCCGAAGTCGGT*
Pten	*TTTGCTAGTGAGTGGAATCCTCT*	*TGTGACAAAAGTGACACAGATCA*
Cip1	*CCTGGTGATGTCCGACCTG*	*CCATGAGCGCATCGCAATC*
Cycb1	*AAGGTGCCTGTGTGTGAACC*	*GTCAGCCCCATCATCTGCG*
Cycd2	*GAGTGGGAACTGGTAGTGTTG*	*CGCACAGAGCGATGAAGGT*

Figure 1S.

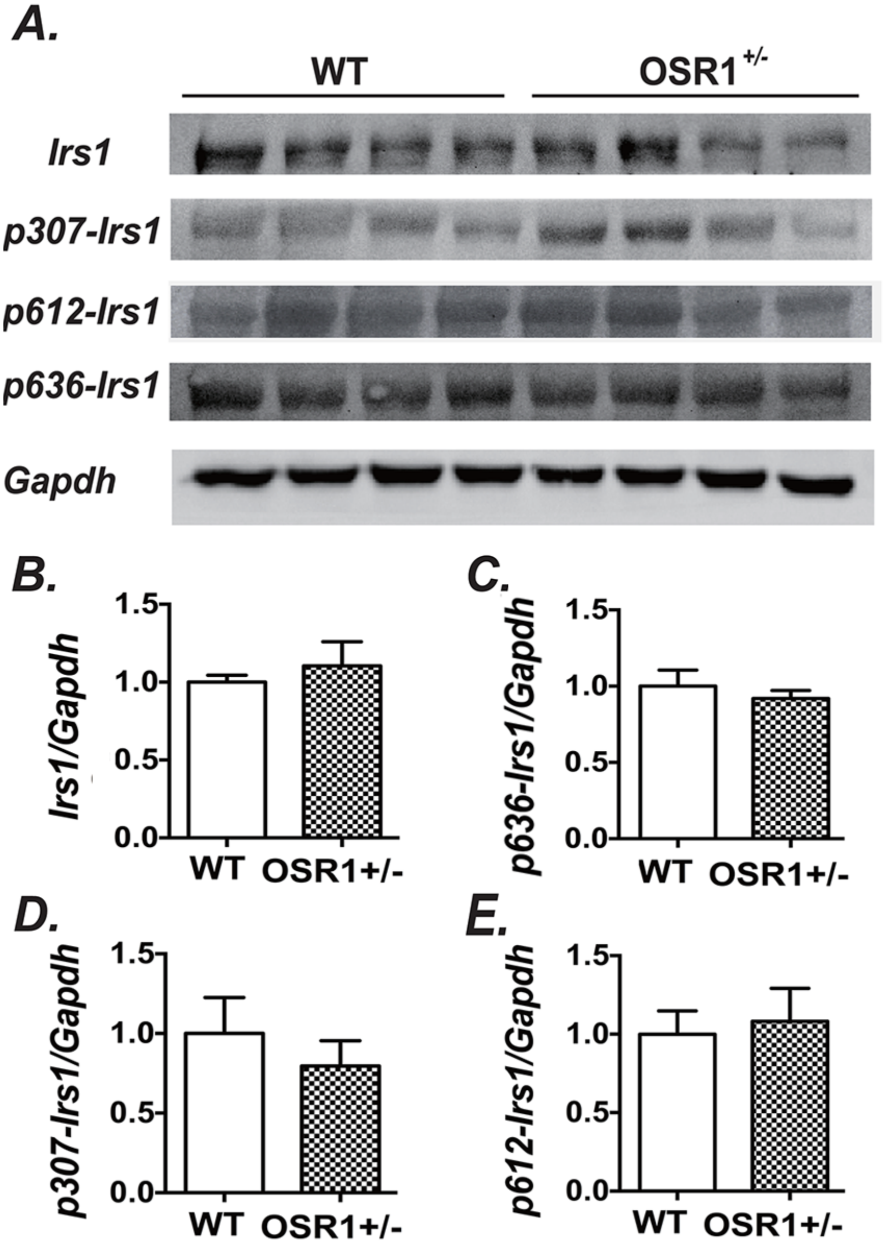

Figure 1S. By protein blotting technique, the insulin signaling genes of *Irs-1* and the phosphorylation of *Irs1* at 307, 612 and 636 were all found to be similar between liver tissues of *Osr1*$^{+/-}$ and wildtype mice.

Data of figures 1S A-E is presented as Mean ± SE, n=4.

Figure 2S.

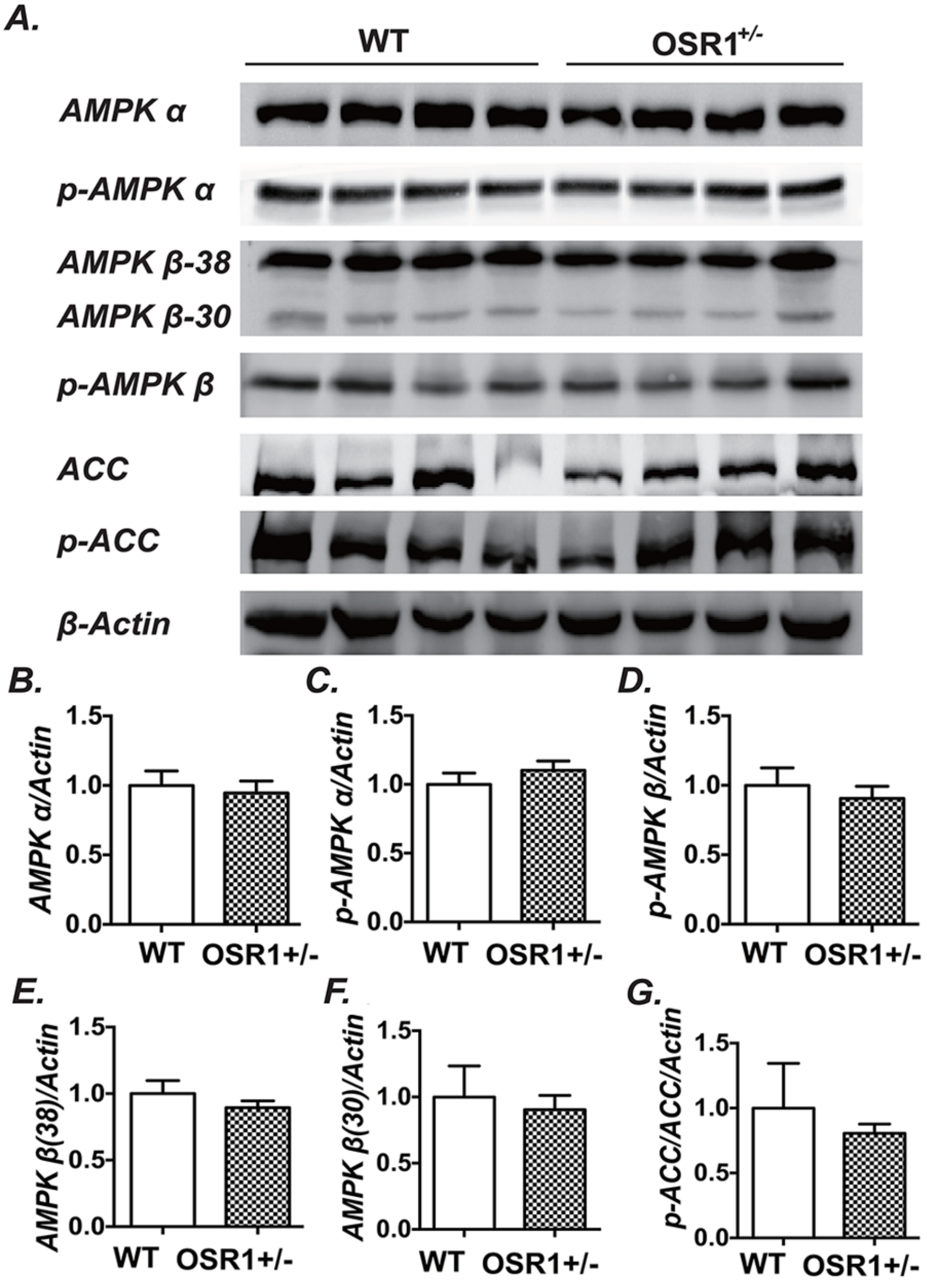

Figure 2S. By protein blotting technique, the genes involved in cellular energy homeostasis, such as *AMPK-α, -β, Acc* and their phosphorylated forms, displayed similar expression levels between *Osr1*$^{+/-}$ and wildtype livers.

Data of figures 2S A-G is presented as Mean ± SE, n=4.
